# PA5201 represses type III secretion system by binding to the P*_exsC_* promoter in *Pseudomonas aeruginosa*

**DOI:** 10.1128/spectrum.04189-25

**Published:** 2026-06-12

**Authors:** Lepeng Wang, Chenyu Shen, Liwen Yin, Yu Zhang, Linyan Zhang, Shujun Liu, Zhihui Cheng, Weihui Wu, Un-Hwan Ha, Shouguang Jin, Yongxin Jin

**Affiliations:** 1 State Key Laboratory of Medicinal Chemical Biology, Key Laboratory of Molecular Microbiology and Technology of the Ministry of Education, Department of Microbiology, College of Life Sciences, Nankai University 12538, Tianjin, China; 2 Department of Biotechnology and Bioinformatics, Korea University 425262 , Sejong, Republic of Korea; Universita degli Studi Roma, Rome, Italy

**Keywords:** *Pseudomonas aeruginosa*, type III secretion system, Tex family protein, PA5201, transcriptional regulation

## Abstract

The type III secretion system (T3SS) is a major virulence determinant in *Pseudomonas aeruginosa*, which is activated by the master regulator ExsA. *exsA* is transcribed from two distinct promoters: its operon promoter P*_exsC_* and its own promoter P*_exsA_*. Here, we identify PA5201, a Tex-family protein, as a novel repressor of T3SS in *P. aeruginosa*. PA5201 directly binds to the P*_exsC_* promoter and represses its activity. Deletion of *PA5201* increased T3SS expression, bacterial cytotoxicity, and bacterial load in a mouse acute pneumonia model, whereas overexpression of *PA5201* decreased T3SS expression. Reporter assays and western blot analysis further revealed that PA5201-mediated repression of P*_exsC_* is independent of ExsA and does not occur at the post-transcriptional level. Electrophoretic mobility shift assay (EMSA) showed that PA5201 binds to the region between −72 and −45 bp relative to the *exsC* start codon. Collectively, this study identifies PA5201 as a novel T3SS repressor and elucidates its molecular mechanism in *P. aeruginosa*.

**IMPORTANCE:**

*Pseudomonas aeruginosa* is an important opportunistic human pathogen responsible for a wide range of infections, particularly in immunocompromised individuals. The type III secretion system (T3SS) plays a critical role in acute *P. aeruginosa* infections. Here, we identify PA5201, a previously uncharacterized Tex-family regulator, as a novel transcriptional repressor of T3SS. We demonstrate that PA5201 directly binds to and represses the P*_exsC_* promoter. This study reveals a new regulator of T3SS in *P. aeruginosa* and clarifies the molecular mechanism of PA5201-mediated T3SS regulation.

## INTRODUCTION

*Pseudomonas aeruginosa*, an opportunistic human pathogen, is a major cause of nosocomial infections, including ventilator-associated pneumonia, bloodstream infections, and chronic lung colonization in cystic fibrosis patients (1). Its virulence and persistence are supported by a diverse arsenal of secretion systems, motility mechanisms, biofilm formation ability, and a complex regulatory network that enables adaptation to environmental and host-derived signals (2). Among these virulence determinants, the type III secretion system (T3SS) functions as a molecular syringe, injecting effector proteins into host cells and playing a key role in colonization and pathogenesis during human and animal infections (3).

In *P. aeruginosa*, ExsA is the master transcriptional activator of T3SS. ExsA controls the expression of all T3SS genes by directly binding to and activating their promoter regions, including that of its own operon (4). ExsA activity is controlled by a partner-switching mechanism involving three other proteins: ExsC, ExsD, and ExsE (5). In the absence of inducing signals, such as low Ca²^+^ and contact with host cells, the secreted repressor ExsE remains cytoplasmic in complex with ExsC, and ExsA is sequestered by ExsD. Whereas in the presence of inducing signals, ExsE is secreted/translocated through the T3SS machinery, which triggers partner switching wherein ExsC preferentially binds ExsD, resulting in free ExsA to activate the T3SS regulon (5). In addition to autoactivation from the operon promoter P*_exsC_*, *exsA* is also driven by its own promoter P*_exsA_* (6). Many known regulators modulate T3SS expression by directly or indirectly influencing the transcription, translation, or activity of ExsA (5). Several regulators control *exsA* expression from P*_exsA_*, including the global virulence factor regulator Vfr (6), the histone-like nucleoid structuring (H-NS) DNA-binding proteins MvaT and MvaU (7), the AraC-family transcriptional factor VqsM (8), and the DNA-binding protein Fis (9). Besides ExsA, MvaT, AmrZ, and PsrA also contribute to T3SS expression by regulating P*_exsC_* promoter activity (10–12).

PA5201 is a transcriptional accessory protein of the Tex family. Tex, initially identified for its role in toxin expression, was first shown to negatively regulate toxin genes *cyaA* and *ptx* in *Bordetella pertussis* (13). Tex also influences pathogen fitness in *Streptococcus pneumoniae* (14). The *tex* gene encodes a transcriptional regulatory protein, and its homologs are found in many bacteria, suggesting a potentially conserved function (13, 14). Although the crystal structure of Tex has been characterized in *P. aeruginosa* (15), its molecular function in this pathogen remains unexplored.

In a previous study, we used a DNA pull-down assay combined with mass spectrometry to identify candidate regulators that bind to and regulate the P*_exsC_* promoter (10). PA5201, a Tex-family protein, was detected in the mass spectrometry data (10). Here, we investigate the role of PA5201 in T3SS and elucidate the molecular mechanisms by which PA5201 represses T3SS in *P. aeruginosa*.

## MATERIALS AND METHODS

### Bacterial strains, plasmids, and culture conditions

The bacterial strains, plasmids, and primers used in this study are listed in Tables S1 and S2. Bacteria were cultured in Luria–Bertani (LB) medium (5 g/L yeast extract, 5 g/L NaCl, and 10 g/L tryptone) or on LB agar plates (LB medium with 15 g/L agar) at 37°C. For plasmid maintenance, antibiotics were used at the following final concentrations (μg/mL): for *P. aeruginosa*, tetracycline (Tc) 50, carbenicillin (Cb) 150; for *Escherichia coli*, Tc10, ampicillin (Amp) 100, kanamycin (Kan) 50. IPTG (isopropyl-β-D-thiogalactopyranoside) was added at 1 mM to induce gene expression. T3SS expression in *P. aeruginosa* was induced with 5 mM EGTA.

### Generation of plasmids and bacterial strains

To complement *PA5201*, the gene was amplified by PCR from PAK genomic DNA using specific primers (Table S2). The PCR product was digested with *Kpn*I and *Hin*dIII and cloned into pUCP20 to generate pUCP20-*PA5201*. pET28a-*PA5201* (*Nco*I-*Xho*I) was constructed similarly.

To generate the *PA5201* deletion construct, upstream and downstream regions of *PA5201* were amplified using primer pairs *PA5201*-UF/UR and *PA5201*-DF/DR, respectively. The fragments were digested and directionally cloned into pEX18Tc to generate pEX18-*PA5201*. To delete *PA5201* in PAK, pEX18-*PA5201* was transferred into PAK via conjugation, and gene deletion was performed using a SacB-based strategy as described (16). To construct P*_tac_*-45-*exsC*-Flag and P*_tac_*-86-*exsC*-Flag vectors, the C-terminal Flag-tagged *exsC* gene, along with its upstream 45-bp or 86-bp untranslated region (UTR), was cloned into the *Eco*RI and *Hin*dIII sites of pMMB67EH. For the P*_tac_*-RBS-*exsC*-Flag vector, the C-terminal Flag-tagged *exsC* gene and its Shine-Dalgarno sequence were amplified and cloned into pMMB67EH at the *Eco*RI and *Hin*dIII sites. To construct the P*_exsC_*-6-*lac*Z and P*_exsC_*_mut_-6-*lac*Z plasmids, a 100 bp fragment upstream of the *exsC* gene with or without deletion of the region between −72 and −45 bp was amplified and cloned into the *Eco*RI-*Bam*HI site of the *lacZ* fused E1553 vector (9, 17).

### β-galactosidase assay

Overnight bacterial cultures were diluted 50-fold into fresh LB medium with or without 5 mM EGTA (T3SS inducing condition) and grown to OD₆₀₀ ≈ 1.0 at 37°C with shaking (200 rpm). One milliliter of culture was centrifuged at 12,000 × *g*, and the pellet was resuspended in 1.5 mL Z buffer (60 mM NaH_2_PO_4_, 60 mM Na_2_HPO_4_, 50 mM β-mercaptoethanol, 10 mM KCl, 1 mM MgSO_4_). One milliliter suspension was used to measure OD₆₀₀. To the remaining 0.5 mL, 10 μL chloroform and 10 μL 0.1% sodium dodecyl sulfate (SDS) were added, followed by vortexing for 15 s. Then, 100 μL of O-nitrophenyl-β-D-galactopyranoside (4 mg/mL) was added, and the reaction proceeded at 37°C. Once the mixture turned light yellow, 500 μL of 1 M Na₂CO₃ was added to stop the reaction, and the reaction time was recorded. After centrifugation, OD₄₂₀ was measured. β-galactosidase activity (Miller units) was calculated as 1,000 × OD₄₂₀/(T × 0.5 × OD₆₀₀), where T is the reaction time (minutes), and 0.5 is the volume (mL) of bacterial suspension used.

### Western blot assay

Overnight cultures were diluted 50-fold into fresh LB medium with or without 5 mM EGTA and grown to OD₆₀₀ ≈ 1.0. Equal numbers of bacterial cells were collected by centrifugation at 12,000 × *g* for 3 min. Supernatant or cell samples were mixed with loading buffer, heated at 99°C for 10 min, and separated by 12% sodium dodecyl sulfate-polyacrylamide gel electrophoresis (SDS-PAGE). After transfer to polyvinylidene difluoride membranes, membranes were probed with rabbit polyclonal anti-ExoS antibody, mouse monoclonal anti-RpoA antibody (RNAP, Abcam), or mouse monoclonal anti-Flag antibody (Sigma) for 1 h at room temperature, followed by horseradish peroxidase-conjugated secondary antibodies for 1 h. RNA polymerase α subunit (RpoA) served as the loading control. Signals were detected using an ECL-plus kit (Millipore) and imaged with ChemiDoc XRS+ (Bio-Rad).

### RNA isolation and real-time qPCR

Overnight *P. aeruginosa* cultures were diluted 50-fold into fresh LB medium and grown to OD₆₀₀ ≈ 1.0. Total RNA was isolated using a Bacterial Total RNA Extraction Kit (Tiangen Biotech, Beijing, China). cDNA was synthesized using PrimeScript Reverse Transcriptase with random primers (TaKaRa, Dalian, China). Real-time qPCR was performed with specific primers (Table S2) and SYBR Premix ExTaq II (TaKaRa, Dalian, China). The *rpsL* gene (encoding 30S ribosomal protein) was used as an internal reference. Reactions were run on a CFX Connect Real-Time PCR System (Bio-Rad), and gene expression was calculated using the 2^−ΔΔCT^ method.

### Mouse acute pneumonia model

The mouse acute pneumonia model was established as previously described with minor modifications (18). Briefly, overnight bacterial cultures were diluted 50-fold into fresh LB medium and grown to OD₆₀₀ ≈ 1.0. Bacterial cells were harvested by centrifugation at 12,000 × *g* for 3 min, washed twice with phosphate-buffered saline (PBS), and adjusted to 5 × 10⁸ CFU/mL in PBS. Six-to-eight-week-old female BALB/c mice were anesthetized by intraperitoneal injection of 90 μL chloral hydrate (7.5%) and intranasally inoculated with 20 μL bacterial suspension per nostril (total 2 × 10⁷ CFU/mouse). At 12 h post-infection, mice were sacrificed, and lungs were isolated and homogenized in 1% protease peptone (Solarbio, Beijing, China). Bacterial loads were quantified by serial dilution and plating.

### Cytotoxicity assay

Bacterial cytotoxicity was assessed by measuring detachment of A549 cells after infection as described (17). Briefly, bacteria at logarithmic phase (OD_600_ ≈ 1.0) were collected, washed with PBS, resuspended in DMEM (Dulbecco’s modified Eagle’s medium, Corning, USA), and used to infect A549 cells at a multiplicity of infection (MOI) of 50. After 3–4 h, the medium was removed, and attached cells were washed twice with PBS and stained with 0.1% crystal violet for 15 min at 37°C. After washing twice with distilled water, the stained crystal violet was dissolved in 200 μL 95% ethanol for 30 min at room temperature, and absorbance was measured at 590 nm.

### Protein purification

*E. coli* BL21 (DE3) harboring pET28a-*PA5201* was subcultured in 200 mL LB medium at 37°C with shaking until OD₆₀₀ reached 0.4–0.6. His-tagged PA5201 expression was induced with 1 mM IPTG for 12–16 h at 16°C. Cells were harvested by centrifugation at 8,000 × *g* for 10 min at 4°C, resuspended in 5 mL lysis buffer (50 mM sodium phosphate, 0.3 M NaCl, pH 8.0), and lysed by sonication on ice. After centrifugation at 12,000 × *g* for 10 min at 4°C, the supernatant was incubated with Ni-NTA resin (Qiagen) for 2 h at 4°C. The resin was washed with lysis buffer containing imidazole (10, 20, 50 mM). PA5201-His was eluted with lysis buffer containing imidazole (100, 200, 300, 400 mM). Purified protein was analyzed by SDS-PAGE with Coomassie brilliant blue staining.

### Electrophoretic mobility shift assays

Electrophoretic mobility shift assay (EMSA) was performed as described (17). DNA fragments were amplified by PCR from PAK genomic DNA using specific primers (Table S2). DNA probes (50 or 20 ng) were incubated with increasing concentrations of purified PA5201-His on ice for 30 min in a 25-μL reaction containing 10% (vol/vol) glycerol, 50 mM Tris-HCl (pH 8.0), and 50 mM KCl. Samples were loaded onto a 12% native polyacrylamide gel in 0.5× TBE buffer (44.5 mM Tris base, 44.5 mM boric acid, 1 mM EDTA, pH 8.0) and electrophoresed on ice at 10 mA for 45–90 min. The gel was stained with 0.5 μg/mL ethidium bromide in 0.5× TBE and imaged with a ChemiDoc XRS+ (Bio-Rad). For the competitive EMSA, 10 ng 6-FAM-labeled P*_exsC_* probe was incubated with 0 or 1 µM purified recombinant PA5201 with or without a 50-fold excess of unlabeled DNA competitors in a 25-μL reaction as above. After electrophoresing as above, the fluorescently labeled bands were visualized with an Amersham Typhoon Scanner.

## RESULTS

### PA5201 binds to P*_exsC_*, the operon promoter of T3SS master activator ExsA

**Fig 1:**
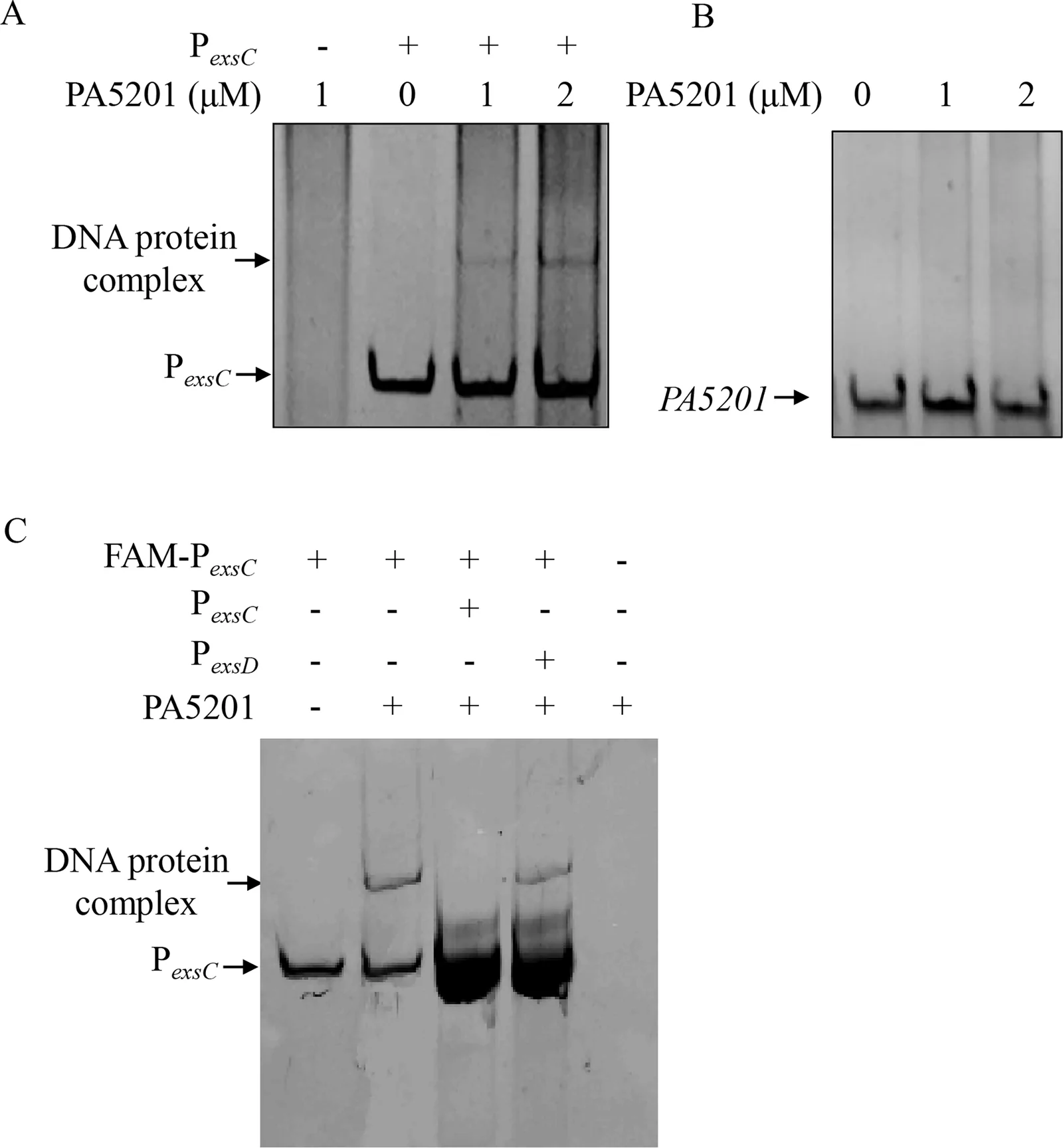
PA5201 binds to the P_*exsC*_ promoter. PA5201 binds to P*_exsC_* (**A**) but not to the *PA5201* DNA fragment (**B**). DNA probe (50 ng) was incubated with 0, 1, or 2 µM PA5201 on ice for 30 min. Shifted bands are indicated by arrowheads. (**C**) 6-FAM-labeled DNA probe (10 ng) was incubated with 0 or 1 µM PA5201 on ice for 30 min. Fifty-fold excess of unlabeled DNA was used as a competitor. Shifted bands are indicated by arrowheads.

In a previous study, DNA pull-down combined with mass spectrometry identified candidate proteins binding to the P*_exsC_* promoter (10). PA5201, a Tex-family protein, was among the detected proteins (10). To confirm binding, EMSA was performed using purified PA5201-His (Fig. S1) and DNA fragments corresponding to the P*_exsC_* promoter. As shown in Fig. 1A and B, the P*_exsC_*, but not a negative control *PA5201* gene fragment, showed a shift upon incubation with PA5021-His. Moreover, the observed shift exhibited a clear dose-dependent pattern. We further performed competitive EMSA using FAM-labeled P*_exsC_*, which also yielded a shifted band upon incubation with PA5201 (Fig. 1C). Binding specificity was confirmed by competition with unlabeled P*_exsC_* DNA, but not with an unrelated DNA fragment corresponding to the promoter region of the *exsD* gene (Fig. 1C). Collectively, these results demonstrate that PA5201 directly binds to the P*_exsC_* promoter in *P. aeruginosa*.

### PA5201 controls ExoS expression and pathogenicity of *P. aeruginosa*

**Fig 2:**
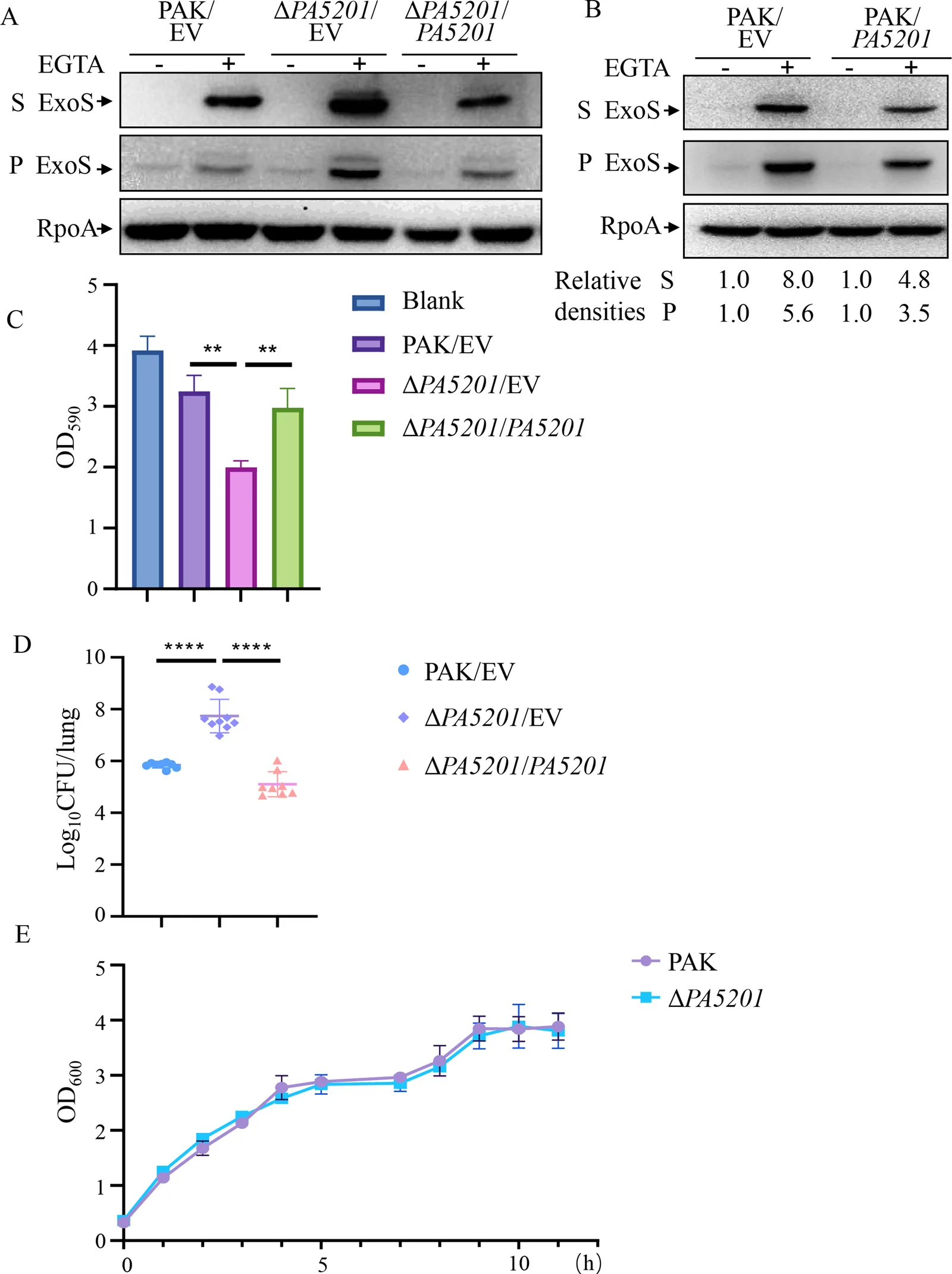
PA5201 controls ExoS expression and bacterial pathogenicity. (**A**) ExoS expression and secretion in PAK/pUCP20, Δ*PA5201*/pUCP20, and Δ*PA5201*/pUCP20-*PA5201*. (**B**) ExoS expression and secretion in PAK/pUCP20 and PAK/pUCP20-*PA5201*. Bacteria were grown to an OD_600_ of approximately 1.0 in LB medium in the presence (+) or absence (−) of 5 mM EGTA. Proteins from supernatants (S) and pellets (P) were separated by 12% SDS-PAGE and detected with anti-ExoS or anti-RpoA antibodies. Band densities in panel **B** were quantified using Image J. Relative densities represent the ratio of ExoS band intensity to RpoA band intensity, with the first lane set to 1. (**C**) Cytotoxicity of indicated strains. A549 cells were infected at an MOI of 50. Three hours post-infection, attached cells were stained with crystal violet, dissolved in 95% ethanol, and quantified at OD_590_. Uninfected cells served as a control. **, *P* < 0.01 (Student’s *t* test). (**D**) Mice were intranasally inoculated with 2 × 10^7^ CFU of the indicated bacterial strains. After 12 h, lungs were homogenized, and bacterial loads were determined. Central line = mean, and the error bar = SD. ****, *P* < 0.0001 (Mann-Whitney test). (**E**) Growth curves of PAK and Δ*PA5201* in LB medium. Error bar = SD.

ExoS, a canonical T3SS effector, is widely used as a readout of T3SS activity (19, 20). To assess the impact of PA5201 on T3SS, we generated a *PA5201* deletion mutant and quantified ExoS expression and secretion by western blot. Strains were grown under T3SS-inducing (5 mM EGTA) or non-inducing conditions (20). As shown in Fig. 2A, *PA5201* deletion markedly increased ExoS expression and secretion compared to wild-type PAK, and complementation with *PA5201* restored ExoS levels to those of PAK. Moreover, ectopic expression of *PA5201* in PAK reduced the ExoS expression and secretion (Fig. 2B), indicating that PA5201 represses T3SS.

Given the role of T3SS in cytotoxicity, we measured bacterial cytotoxicity using a crystal violet assay. We infected human adenocarcinoma alveolar epithelial lung cells A549 with PAK, Δ*PA5201*, and the complemented strain Δ*PA5201*/*PA5201*. Detached cells caused by cytotoxicity were washed away, and the remaining cells were quantified by crystal violet staining. As shown in Fig. 2C, *PA5201* deletion significantly increased cytotoxicity, which was restored to wild-type levels by complementation (Fig. 2C), consistent with altered T3SS activity.

The link between T3SS and pathogenicity led us to examine PA5201’s role in virulence using a murine acute pneumonia model (21). Mice were infected intranasally with wild-type PAK, Δ*PA5201* mutant, or the complemented strain Δ*PA5201*/*PA5201*. Bacterial loads in lungs at 12 h post-infection were significantly higher for Δ*PA5201* than for PAK or the complemented strain (Fig. 2D), indicating enhanced virulence upon *PA5201* loss. This was not due to increased growth rate, as Δ*PA5201* and PAK showed similar growth in LB medium (Fig. 2E). Together, these molecular, cellular, and animal model results identify PA5201 as a negative regulator of T3SS in *P. aeruginosa*.

### PA5201 represses P*_exsC_* promoter activity independently of ExsA protein

**Fig 3:**
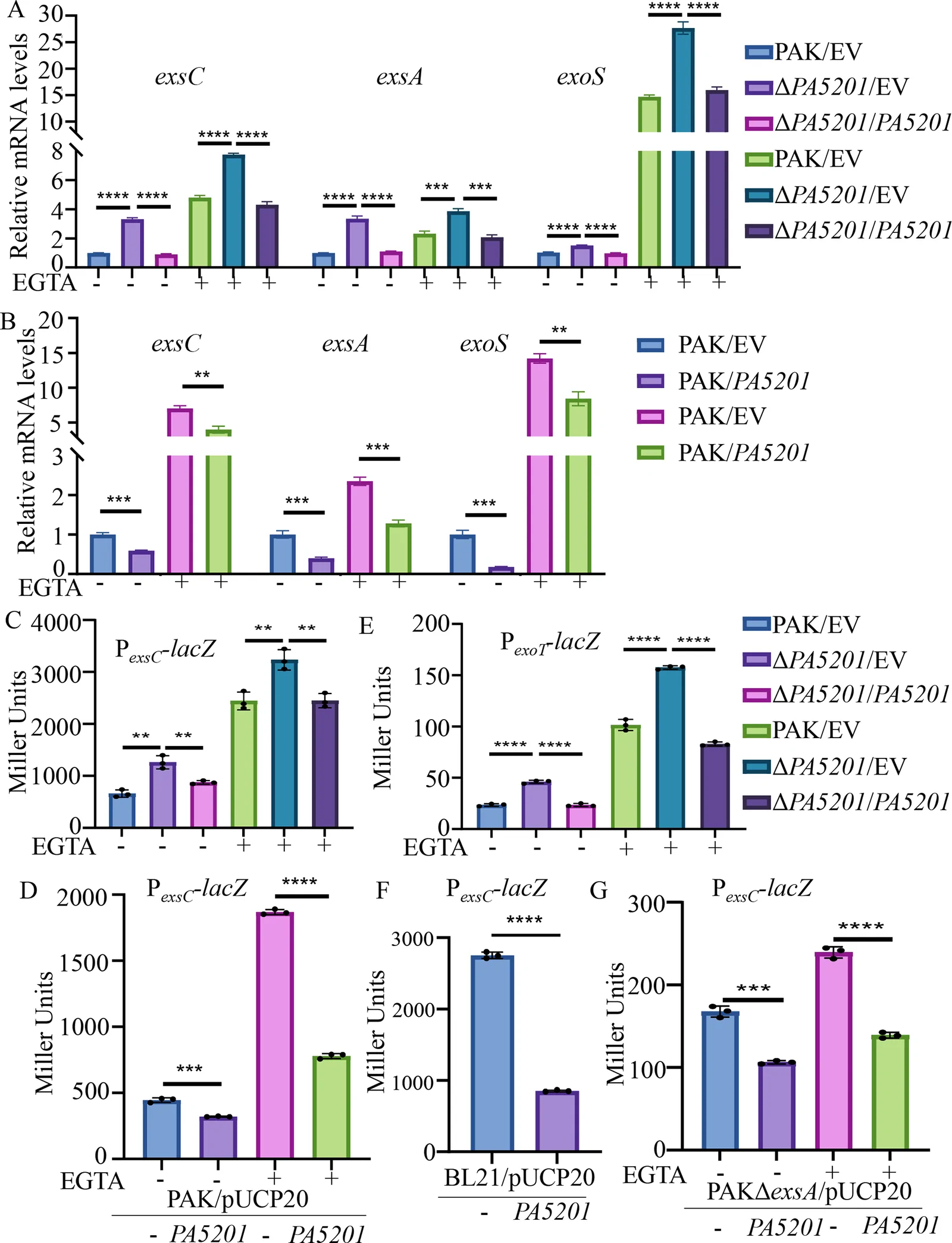
PA5201 represses T3SS independently of ExsA. (**A and B**) Relative mRNA levels of *exsC*, *exsA*, and *exoS* in the indicated bacterial strains under T3SS-inducing (+) and non-inducing (−) conditions. *rpsL* was used as an internal control. (**C–G**) β-galactosidase assays of indicated bacterial strains carrying P*_exsC_-lac*Z (**C, D, F, and G**) or P*_exoT_-lac*Z (**E**) reporter were grown to an OD_600_ of 1.0 in LB with (+) or without (− or in F) 5 mM EGTA. Assays were performed in triplicate; error bars = SD. **, *P* < 0.01; ***, *P* < 0.001; ****, *P* < 0.0001 (Student’s *t* test).

To further investigate PA5201’s effect on T3SS transcription, real-time qPCR was used to measure mRNA levels of *exsC*, *exsA*, and *exoS* in wild-type PAK, Δ*PA5201*, and complemented strains. As shown in Fig. 3A, all three genes were significantly upregulated in Δ*PA5201* compared to PAK and the complemented strain. Overexpression of PA5201 further reduced mRNA levels of these genes in PAK (Fig. 3B). To confirm transcriptional changes, a P*_exsC_-lacZ* transcriptional fusion was introduced into PAK, Δ*PA5201*, and complemented strains. β-galactosidase activity was significantly higher in Δ*PA5201* than in PAK or the complemented strain under both T3SS-inducing and non-inducing conditions (Fig. 3C). Overexpression of *PA5201* in PAK reduced β-galactosidase activity (Fig. 3D). A similar pattern was observed for P*_exoT_-lac*Z (Fig. 3E), indicating that PA5201 suppresses T3SS at the transcriptional level.

Given PA5201’s binding to P*_exsC_* and its effect on T3SS transcription, we asked whether PA5201 directly represses P*_exsC_*. A reporter assay in *E. coli* BL21 (DE3) showed that *PA5201* expression significantly reduced P*_exsC_-lac*Z activity compared to the empty vector (Fig. 3F), indicating direct repression. To further validate direct repression independent of ExsA protein, we measured P*_exsC_-lac*Z activity in a PAKΔ*exsA* background. β-galactosidase activity was significantly lower upon PA5201 expression in PAKΔ*exsA* (Fig. 3G), confirming that PA5201 directly regulates the P*_exsC_* promoter independently of ExsA protein.

### Repression of *exsC* does not occur at the post-transcriptional level

**Fig 4:**
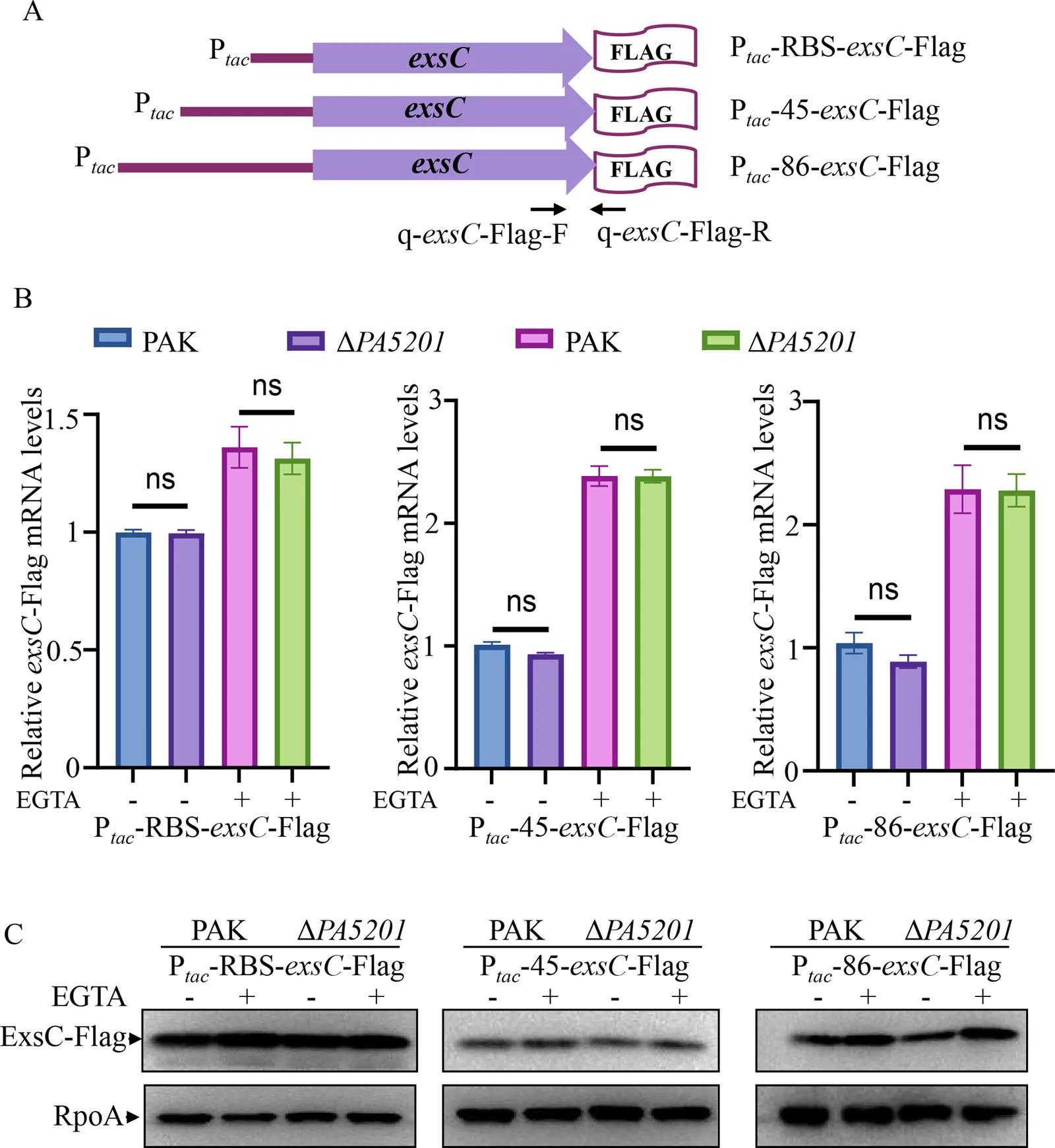
Repression of ExsC does not occur at the post-transcriptional level. (**A**) Schematic of *exsC*-Flag constructs in pMMB67EH. (**B**) Relative *exsC-*Flag mRNA levels in indicated strains under T3SS-inducing (+) and non-inducing (−) conditions (1 mM IPTG added). *rpsL* was an internal control. ns, not significant (Student’s *t* test). (**C**) ExsC-Flag protein levels in the indicated strains. Bacteria were grown to an OD_600_ of 1.0 with 1 mM IPTG and with (+) or without (−) 5 mM EGTA. Equivalent cell numbers were analyzed by western blot with anti-Flag or anti-RpoA antibody. Data are from three independent experiments.

PA5201 contains a C-terminal S1 domain, an RNA-binding motif found in Tex-family proteins (13). If PA5201 binds to the 5′ UTR of *exsC* mRNA and represses its translation at the post-transcriptional level, the resulting decrease in ExsC protein will prevent it from binding ExsD and releasing ExsA, thereby also leading to repression of the T3SS. To test whether PA5201 regulates *exsC* post-transcriptionally, we constructed three vectors in which C-terminal Flag-tagged *exsC* with varying 5′ UTR lengths was driven by P*_tac_* promoter (Fig. 4A). These constructs were introduced into wild-type PAK and the Δ*PA5201* mutant. qPCR showed no significant difference in *exsC-*Flag mRNA levels between the two strains (Fig. 4B). Western blot also showed similar ExsC-Flag protein levels (Fig. 4C), indicating that PA5201 does not regulate *exsC* post-transcriptionally.

### PA5201 binds to the −72 to −45 bp region relative to the *exsC* translational start codon and inhibits transcription

**Fig 5:**
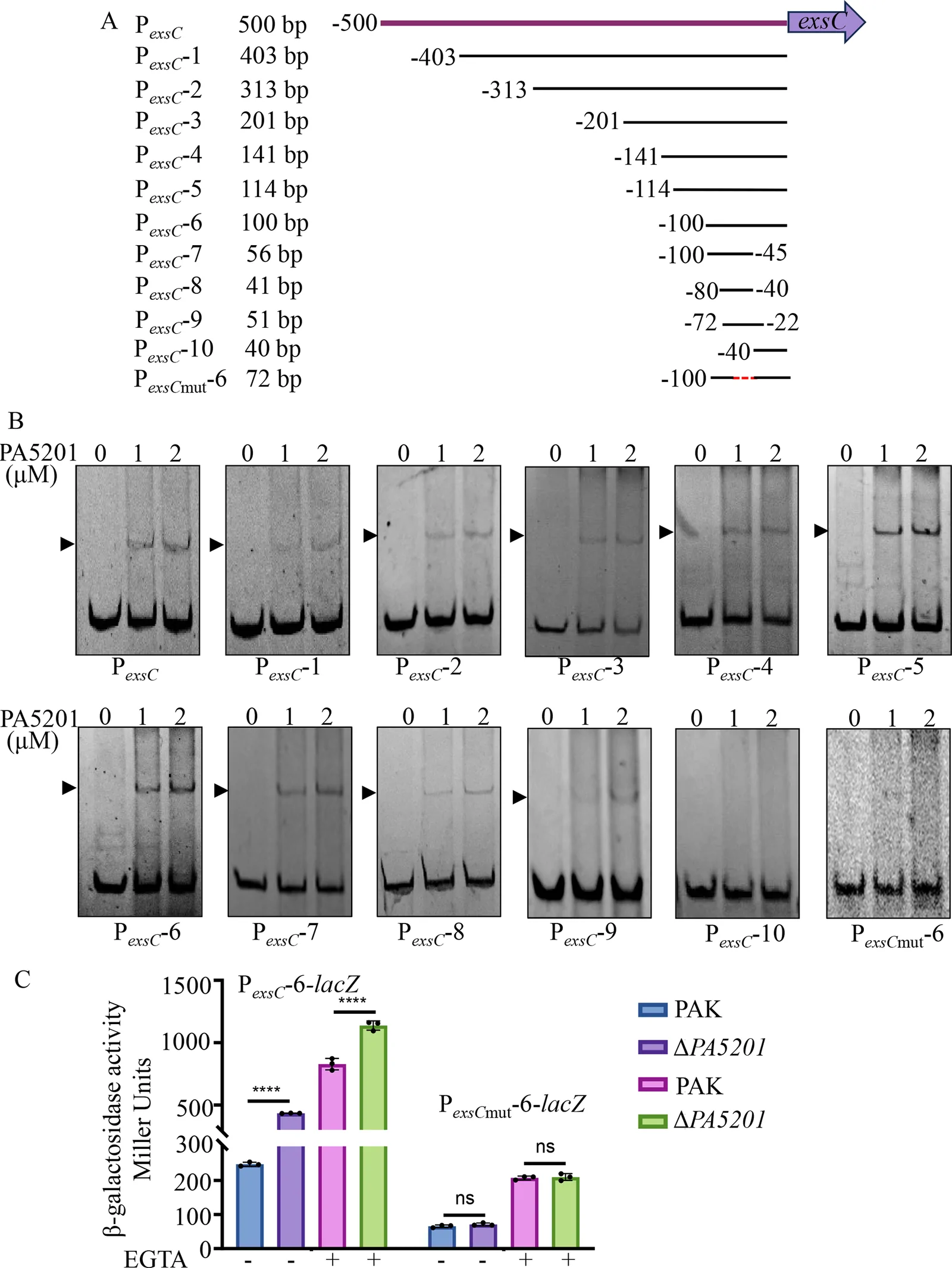
PA5201 binds to the −72 to −45 bp region to repress P*_exsC_* activity. (**A**) , Diagram of DNA probes within the P*_exsC_*. Numbers indicate positions relative to the *exsC* start codon. (**B**) DNA fragments were incubated with 0, 1, or 2 µM PA5201 on ice for 30 min. Shifted bands were indicated by arrowheads. (**C**) β-galactosidase assays of PAK and Δ*PA5201* carrying P*_exsC_*-6-*lac*Z or P*_exsC_*_mut_-6-*lac*Z grown to an OD_600_ of 1.0 in LB with 0 (−) or 5 mM (+) EGTA. Assays in triplicate; error bars = SD. ns, not significant; ****, *P* < 0.0001 (Student’s *t* test).

The above data show that PA5201 directly binds to and inhibits the P*_exsC_* independently of ExsA. To localize the binding site, we generated a series of DNA fragments within the P*_exsC_* promoter (Fig. 5A) for EMSA. A fragment containing the 100 bp upstream region still showed a shift with PA5201-His (Fig. 5B). Four overlapping fragments, P*_exsC_*-7 (−100 to −45), P*_exsC_*-8 (−80 to −40), P*_exsC_*-9 (−72 to −22), and P*_exsC_*-10 (−40 to -1) were tested. P*_exsC_*-7, P*_exsC_*-8, and P*_exsC_*-9, but not P*_exsC_*-10, showed shifts, suggesting the binding site lies within the −72 to −45 overlap. Deletion of this region (P*_exsC_*_mut_-6) abolished binding (Fig. 5B), confirming it is the critical PA5201-binding site.

To assess the role of this site in PA5201-mediated repression, we constructed transcriptional fusion reporters with P*_exsC_*-6 and P*_exsC_*_mut_-6 (P*_exsC_*-6-*lacZ* and P*_exsC_*_mut_-6-*lacZ*). β-galactosidase activities were significantly higher in Δ*PA5201/*P*_exsC_*-6-*lacZ* than in PAK under both T3SS-inducing and non-inducing conditions (Fig. 5C). In contrast, removal of the binding site eliminated differences between PAK and Δ*PA5201* (Fig. 5C). These results confirm that PA5201 binds to −72 to −45 bp and represses the P*_exsC_*.

## DISCUSSION

PA5201 is a Tex-family protein with previously unknown function in *P. aeruginosa*. Here, we identify PA5201 as a novel repressor of T3SS in *P. aeruginosa*. We show that PA5201 directly binds to the P*_exsC_* promoter to repress *exsA* transcription and subsequent T3SS expression. PA5201 contains an N-terminal helix-turn-helix (HtH) domain, a YqgF-homologous domain, a helix-hairpin-helix (HhH) domain, and a C-terminal S1 domain (15). A previous structural study showed that Tex binds nucleic acids with successive preference for single-stranded RNA, double-stranded DNA, double-stranded RNA, and single-stranded DNA (15). If PA5201 bound to the 5′ UTR of *exsC* mRNA to repress expression post-transcriptionally, reduced ExsC would affect ExsD sequestration and ExsA activity. However, our western blot data show that PA5201 does not regulate *exsC* post-transcriptionally.

PA5201 is a Tex-family transcriptional accessory protein with a C-terminal S1 motif (https://www.pseudomonas.com/). Our study shows that it directly binds DNA to repress transcription. S1-motif proteins have been reported to bind DNA (14, 15, 22); Tex-family proteins are known to bind nucleic acids and modulate gene expression at the transcriptional level (13, 14). For example, Tex binds chromosomal DNA and RNA to influence fitness in *S. pneumoniae* (14), and negatively regulates toxin expression in *B. pertussis* (13). While our data show no post-transcriptional regulation of *exsC* by PA5201, the S1 motif preferentially binds single-stranded RNA (15), suggesting possible post-transcriptional roles warranting further study.

**Fig 6:**
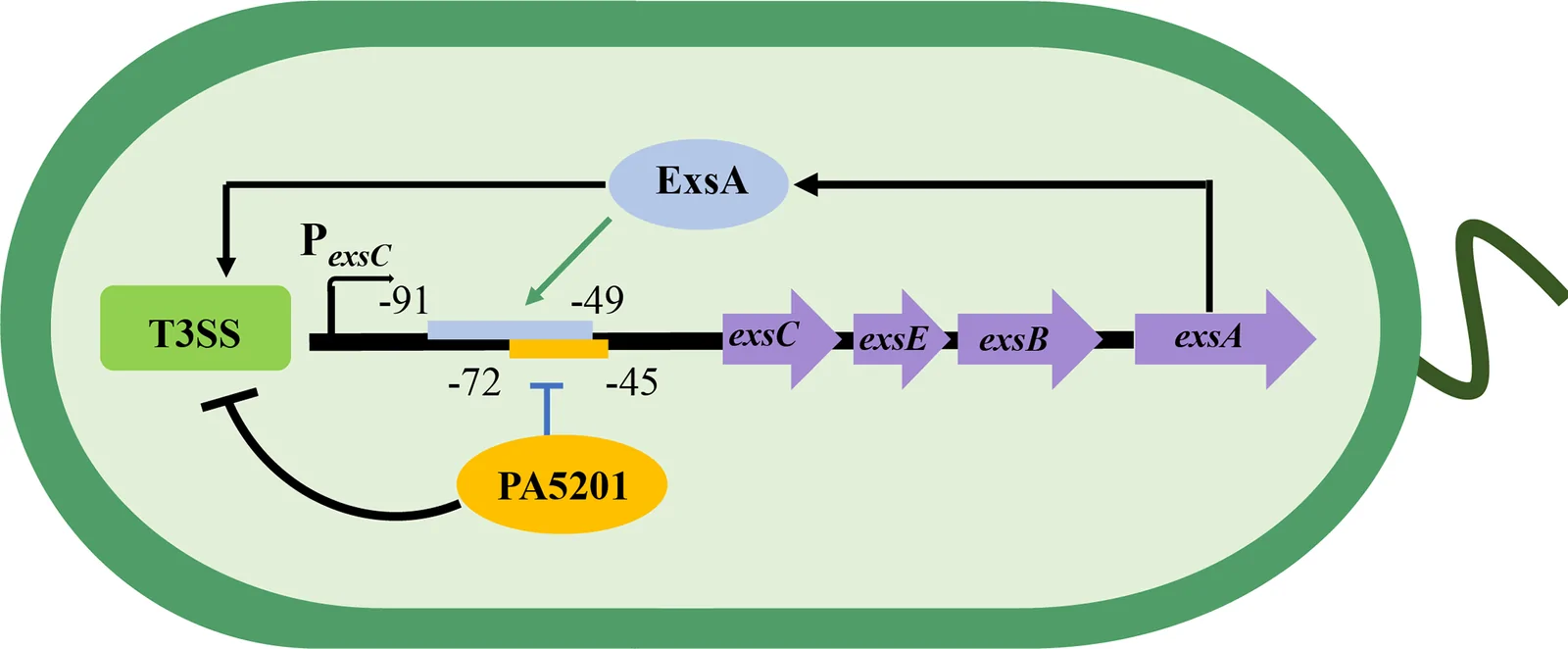
Proposed model of PA5201’s role in *P. aeruginosa*. Numbers indicate binding sites relative to the *exsC* start codon. ExsA binding site from https://www.pseudomonas.com/ and reference 20.

Previous studies showed that the H-NS family protein MvaT binds and inhibits P*_exsC_* (10), the TetR-family transcriptional regulator PsrA binds and stimulates P*_exsC_* promoter activity (11), and AmrZ binds and represses P*_exsC_* in *P. aeruginosa* (12). In this study, we find that PA5201 binds to −72 to −45 bp relative to the *exsC* start codon to repress its transcription. Notably, this site overlaps with the ExsA binding site (−91 to −49 bp) (Fig. 6) (https://www.pseudomonas.com/) (20). The persistence of PA5201-mediated repression in the absence of *exsA* indicates direct repression, possibly by interfering with RNAP binding or promoter escape. Interestingly, PA5201 copurifies with RNA polymerase, suggesting a role in regulating RNA polymerase activity (15). Interference with ExsA occupancy by PA5201 cannot be excluded at present.

Interestingly, our previous transcriptome analysis revealed that *psrA* was upregulated in *P. aeruginosa* during murine acute pneumonia infection (23), which may contribute to the elevated expression of the type III secretion system in acute infection. At the same time, we also observed that the expression of *PA5201* was induced under the same conditions (23). This is not the first instance in *P. aeruginosa* where a T3SS repressor is upregulated by T3SS-inducing signals. For example, PtrA (*Pseudomonas* type III repressor A) was also found to be elevated during infection in mouse burn wound models (24). Together, these findings suggest that *P. aeruginosa* has evolved sophisticated regulatory mechanisms to tightly modulate the energy-costly T3SS machinery in response to specific host environments.

This study reveals PA5201 as a repressor of T3SS in *P. aeruginosa*. Our work elucidates the molecular mechanism of PA5201-mediated T3SS repression. Further investigation into the global role of PA5201 may provide insights for new therapeutic strategies to control *P. aeruginosa* infections.

## SUPPLEMENTAL MATERIAL

Fig. S1 spectrum.04189-25-s0001.tif SDS PAGE of PA5201.

Table S1 spectrum.04189-25-s0002.docx Strains and plasmids.

Table S2 spectrum.04189-25-s0003.doc Primers.

ASM does not own the copyrights to Supplemental Material that may be linked to, or accessed through, an article. The authors have granted ASM a non-exclusive, world-wide license to publish the Supplemental Material files. Please contact the corresponding author directly for reuse.
