# Supplementary figures and images for: PA5201 represses type III secretion system by binding to the PexsC promoter in Pseudomonas aeruginosa
Source: Microbiol Spectr. 2026 Jun 12;14(7):e04189-25. doi: 10.1128/spectrum.04189-25 (PMC13340108; doi:10.1128/spectrum.04189-25)

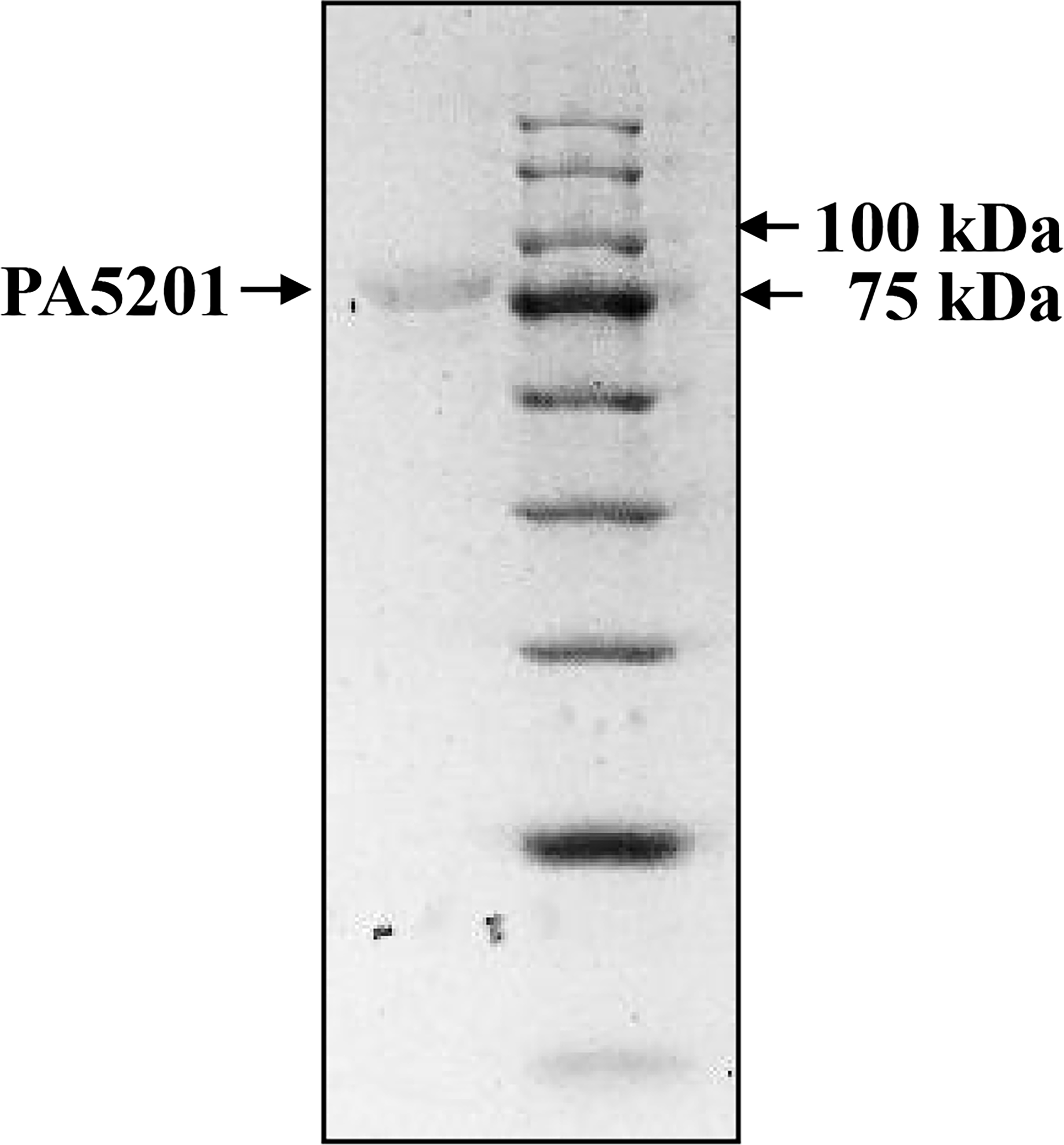

Supplement: Fig. S1 — SDS PAGE of PA5201. [file spectrum.04189-25-s0001.tif]
