# Supplementary material for: PA5201 represses type III secretion system by binding to the PexsC promoter in Pseudomonas aeruginosa
Source: Microbiol Spectr. 2026 Jun 12;14(7):e04189-25. doi: 10.1128/spectrum.04189-25 (PMC13340108; doi:10.1128/spectrum.04189-25)
Supplement: Table S1 — Strains and plasmids. [file spectrum.04189-25-s0002.docx]

**Table S1.** Bacterial strains and plasmids used in this study.

| Strains or plasmids | Description | Source or reference |
| --- | --- | --- |
| **strains** |  |  |
| DH5α | F^̶^ ϕ 80d*lacZ*∆M15 *endA1 recA1 hsdR17*(r_K_^̶^ m_K_^+^) *supE44 thi-1 relA1* ∆(*lacZYA-argF*)*U169 gyrA96 deoR* | TransGen |
| S17-1 | RP4-2 Tc::Mu Km::Tn*7* Tp^r^ Sm^r^ Pro Res^̶^ Mod^+^ | Stratagene |
| BL21 (DE3) | F^-^ *ompT* *hsdSB (rB-, mB-) gal dcm* (DE3) | Invitrogen |
| PAK | Wild-type *P. aeruginosa* strain | David Bradley |
| Δ*PA5201* | PAK with *PA5201* gene deleted | This study |
| PAKΔ*exsA* | PAK with *exsA* gene deleted | [1] |
|  |  |  |
| **Plasmids** |  |  |
| pUCP20 | Shuttle vector between *E. coli* and *P. aeruginosa*; Amp^r^ | [2] |
| pUCP20-*PA5201* | His-tagged *PA5201* gene from PAK in pUCP20; Amp^r^ | This study |
| pMMB67EH | Shuttle vector pMMB67EH between *E. coli* and *P. aeruginosa*; Amp^r^ | ATCC |
| pEX18Tc | Gene knockout vector; Tc^r^ | [3] |
| pEX18-*PA5201* | *PA5201* gene deletion on pEX18Tc; Tc^r^ | This study |
| P*_exsC_*-*lacZ* | *exsC* promoter fused to promoterless *lac*Z on pDN19*lac*ZΩ; Sp^r^, Sm^r^, Tc^r^ | [4] |
| P*_exoT_*-*lacZ* | *exoT* promoter fused to promoterless *lac*Z on pDN19*lac*ZΩ; Sp^r^, Sm^r^, Tc^r^ | [5] |
| P*_exsC_*-6-*lacZ* | *exsC* promoter fused to promoterless *lac*Z on promoterless pUCP20 *lac*ZΩ; Amp^r^ | This study |
| P*_exsC_*_mut_-6-*lacZ* | *exsC* promoter without *PA5201* binding region fused to promoterless *lac*Z on promoterless pUCP20; Amp^r^ | This study |
| P*_tac_*-RBS-*exsC*-Flag | RBS-*exsC-*Flag driven by an inducible *tac* promoter in pMMB67EH; Amp^r^ | This study |
| P*_tac_*-45-*exsC-*Flag | *exsC-*Flag with 45 bp UTR driven by an inducible *tac* promoter in pMMB67EH; Amp^r^ | This study |
| P*_tac_*-86-*exsC*-Flag | *exsC*-Flag with 86 bp UTR driven by an inducible *tac* promoter in pMMB67EH; Amp^r^ | This study |
| pET28a | expression vector, Kan^r^ | Novagen |
| pET28a-*PA5201* | *PA5201* gene cloned into pET28a expression vector, Kan^r^ | This study |

1. Yin, L., et al., *MvaT binds to the P(exsC) promoter to repress the type III secretion system in Pseudomonas aeruginosa.* Front Cell Infect Microbiol, 2023. **13**: p. 1267748.

2. West, S.E., et al., *Construction of improved Escherichia-Pseudomonas shuttle vectors derived from pUC18/19 and sequence of the region required for their replication in Pseudomonas aeruginosa.* Gene, 1994. **148**(1): p. 81-6.

3. Schweizer, H.P., *Allelic exchange in Pseudomonas aeruginosa using novel ColE1-type vectors and a family of cassettes containing a portable oriT and the counter-selectable Bacillus subtilis sacB marker.* Mol Microbiol, 1992. **6**(9): p. 1195-204.

4. Deng, X., et al., *Fis Regulates Type III Secretion System by Influencing the Transcription of exsA in Pseudomonas aeruginosa Strain PA14.* Front Microbiol, 2017. **8**: p. 669.

5. Ha, U. and S. Jin, *Growth phase-dependent invasion of Pseudomonas aeruginosa and its survival within HeLa cells.* Infect Immun, 2001. **69**(7): p. 4398-406.
