# Supplementary material for: PA5201 represses type III secretion system by binding to the PexsC promoter in Pseudomonas aeruginosa
Source: Microbiol Spectr. 2026 Jun 12;14(7):e04189-25. doi: 10.1128/spectrum.04189-25 (PMC13340108; doi:10.1128/spectrum.04189-25)
Supplement: Table S2 — Primers. [file spectrum.04189-25-s0003.doc]

**Table S2**. Primers used in this study.

| Primer^a^ | Sequence 5’-3’ | Use |
| --- | --- | --- |
| *PA5201*-F | GGGGTACCCCGTTTTCGTTCGTTTTACCAGG | pUCP20-*PA5201* |
| *PA5201-R* | CCAAGCTTTCAATGGTGATGGTGATGATGCTTCTTCTTCAGTTGCTTGGC |
| *PA5201*-UF | CGGAATTCTTGCGGGTAAGACGACGCG | pEX18-*PA5201* |
| *PA5201*-UR | GCTCTAGATGATCCACCTGGTAAAACGAACG |
| *PA5201*-DF | GCTCTAGACGGAGCCGTCCATGAGCG |
| *PA5201*-DR | CCAAGCTTGCGATCGGGATGCTCTCCTC |
| pET28a-*PA5201-*F | AACTTTAAGAAGGAGATATACCATGGACAGCATCAACACCCGTATCGCCG | pET28a-*PA5201* |
| pET28a- *PA5201*-R | TGGTGGTGGTGGTGGTGCTCGAGCTTCTTCTTCAGTTGCTTGGCGTTGGC |
| P*_tac_*-RBS-*exsC*-Flag-F | CCGGAATTCAAGGAGATATACCATGGATTTAACGAGCAAGG | P*_tac_*-RBS-*exsC*-Flag |
| P*_tac_*-RBS-*exsC*-Flag-R | CCCAAGCTTTCACTTATCGTCGTCATCCTTGTACGACGATAAGTGAAAGCTTGGG |
| P*_tac_*-45-*exsC*-Flag-F | CCGGAATTCTGCATAGCCCGGTGCTAGCATG | P*_tac_*-45-*exsC*-Flag |
| P*_tac_*-45-*exsC*-Flag-R | CCCAAGCTTTCACTTATCGTCGTCATCCTTGTAATCAACCCTCATGCCGACCG |
| P*_tac_*-86-*exsC*-Flag-F | CCGGAATTCCTTTTTTCTTAAAAGAAAAGTCTCTCAGTGACAAAAGCGA | P*_tac_*-86-*exsC*-Flag |
| P*_tac_*-86-*exsC*-Flag-R | CCCAAGCTTTCACTTATCGTCGTCATCCTTGTAATCAACCCTCATGCCGACCG |
| P*_exsC_*-6-*lacZ*-F | CCGGAATTCGCCACAGCGATGTGGCTTTTTTCTTAAAAG | P*_exsC_*-6-*lacZ* |
| P*_exsC_*-6-*lacZ*-R | CGCGGATCCGGGGGCGCCTCCTAAAGC |
| P*_exsC_*_mut_-6-*lacZ*-F | CCGGAATTCGCCACAGCGATGTGGCTTTTTTCTTAAAAG | P*_exsC_*_mut_-6-*lacZ* |
| P*_exsC_*_mut_-6-*lacZ*-R | CGCGGATCCGGGGGCGCCTCCTAAAGCTCAGCGCATGCTAGCACCGGGCTATGCTTTTAAGAAAAAAGCCAC |
| EMSA |  |  |
| P*_exsC_*-F^b^ | GCGCTTGGCAAGACCTCCG | P*_exsC_* |
| P*_exsC_*-R | TGGGGGCGCCTCCTAAAGC |
| P*_exsD_*-F | GCTGATGCTCTTCGCGTTCAGTC | P*_exsD_* |
| P*_exsD_*-R | TTCTCTGCCTTGGCTTCCTCACTAC |
| P*_exsC_*-1-F | GTGCCTTCGAGAGCCGCAACG | P*_exsC_*-1 |
| P*_exsC_*-2-F | GCCAGGGCGAATCGCAGG | P*_exsC_*-2 |
| P*_exsC_*-3-F | GGACGTCCTGCAGCTCATCCAG | P*_exsC_*-3 |
| P*_exsC_*-4-F | GGCCGGAGTGGTCTGAGCC | P*_exsC_*-4 |
| P*_exsC_*-5-R | GCGGGAGGAAAAGGCCACAG | P*_exsC_*-5 |
| P*_exsC_*-6-R | CCACAGCGATGTGGCTTTTTTCTTAAAAGAAAAG | P*_exsC_*-6 |
| P*_exsC_*-7-F | CCACAGCGATGTGGCTTTTTTCTTAAAAGAAAAAGTCTCTCAGTGACAAAAGCGATG | P*_exsC_*-7 |
| P*_exsC_*-7-R | CATCGCTTTTGTCACTGAGAGACTTTTCTTTTAAGAAAAAAGCCACATCGCTGTGG |
| P*_exsC_*-8-F | TCTTAAAAGAAAAGTCTCTCAGTGACAAAAGCGATGCATAGCTATGCATCGCTTTTGTCACTGAGAGACTTTTCTTTTAAGA | P*_exsC_*-8 |
| P*_exsC_*-8-R | CTATGCATCGCTTTTGTCACTGAGAGACTTTTCTTTTAAGA |  |
| P*_exsC_*-9-F | GAAAAGTCTCTCAGTGACAAAAGCGATGCATAGCCCGGTGCTAGCATGCGC | P*_exsC_*-9 |
| P*_exsC_*-9-R | GCGCATGCTAGCACCGGGCTATGCATCGCTTTTGTCACTGAGAGACTTTTC |
| P*_exsC_*-10-F | GCCCGGTGCTAGCATGCGCTGAGCTTTAGGAGGCGCCCCC | P*_exsC_*-10 |
| P*_exsC_*-10-R | GGGGGCGCCTCCTAAAGCTCAGCGCATGCTAGCACCGGGC |
| P*_exsC_*_mut_-6-F | CCACAGCGATGTGGCTTTTTTCTTAAAAGCATAGCCCCCGGTGCTAG | P*_exsC_*_mut_-6 |
| P*_exsC_*_mut_-6-R | GGGGGCGCCTCCTAAAGCTCAGCGCATGCTAGCACCGGGCTATGCTTTTAAGAAAAAAGCCAC |
| qPCR primer |  |  |
| q-*exoS-*F | GCATATTCAATCGCTTCAG | qPCR of *exoS* |
| q-*exoS-*R | CCTCAATCTGTCCCAAAC |
| q-*exsA-*F | GCTATGTCGTAAGTACCA | qPCR of *exsA* |
| q-*exsA-*R | GAAGCCTTGTAGAAACTG |
| q-*exsC-*F | CAGCTTCAACCGCCATTG | qPCR of *exsC* |
| q-*exsC-*R | CGCATACAACTGGACCTTG |
| q-*rpsL-*F | CAAAACTGCCCGCAACGT | qPCR of *rpsL* |
| q-*rpsL-*R | TTTCGGCGTGGTGGTGTAT |
| q-*exsC*-Flag*-*F | CGAGGGGATCTTTCGCCAG | qPCR of *exsC*-Flag |
| q-*exsC*-Flag *-*R | ATCGTCGTCATCCTTGTAATCAACC |

a: F: forward; R, reverse; U, upstream of specific gene; D, downstream of specific gene; q, qPCR.

b: This primer was also labelled with 6-FAM and used to amplify the fragment for competitive EMSA.
